# Metagenomic data of free cyanide and thiocyanate degrading bacterial communities

**DOI:** 10.1016/j.dib.2017.06.049

**Published:** 2017-07-04

**Authors:** Lukhanyo Mekuto, Seteno K.O. Ntwampe, John B.N. Mudumbi, Enoch A. Akinpelu, Maxwell Mewa-Ngongang

**Affiliations:** Bioresource Engineering Research Group (BioERG), Department of Biotechnology, Cape Peninsula University of Technology, P.O. Box 652, Cape Town, 8000 South Africa

**Keywords:** Cyanide degrading organisms, Thiocyanate degrading organisms, Metagenomics, 16S rRNA gene

## Abstract

The data presented in this article contains the bacterial community structure of the free cyanide (CN^-^) and thiocyanate (SCN^-^) degrading organisms that were isolated from electroplating wastewater and synthetic SCN^-^ containing wastewater. PCR amplification of the 16S rRNA V1-V3 regions was undertaken using the 27F and 518R oligonucleotide primers following the metacommunity DNA extraction procedure. The PCR amplicons were processed using the illumina® reaction kits as per manufacturer׳s instruction and sequenced using the illumina® MiSeq-2000, using the MiSeq V3 kit. The data was processed using bioinformatics tools such as QIIME and the raw sequence files are available via NCBI׳s Sequence Read Archive (SRA) database.

**Specifications Table**

**Table:** 

Subject area	Biology, Microbial ecology, Biodiversity
More specific subject area	Metagenomics
Type of data	Table
How data was acquired	Sequencing was conducted on an Illumina® MiSeq-2000, using a MiSeq V3 (600 cycle) kit following the procedures developed at Inqaba Biotech (Pretoria, South Africa) (www.inqababiotec.co.za).
Data format	Raw data
Experimental factors	The flanking regions of the 16S rRNA gene (V1-V3) were PCR amplified using the 27F and 518R oligonucleotide primers.
Experimental features	Cyanide degrading organisms (CDOs) were isolated in electroplating wastewater. Since the CDOs were unable to degrade SCN^-^, a gravimetric technique was employed in synthetic wastewater containing SCN^-^ outside the BioERG laboratory. Metacommunity DNA was extracted from both the CDOs and TDOs for sequencing.
Data source location	BioERG laboratory, Cape Town, South Africa (33.9324°S, 18.6406°E) Electroplating facility, Cape Town, South Africa (33.9708°S, 18.5780°E)
Data accessibility	The accession numbers of the sequence data are publicly available on a public repository (http://hdl.handle.net/11189/5110) and are also embedded within Supplementary Table 1 and 2.

**Value of the data**

• This research data provides crucial information on the bacterial community structure and differences between the CDOs and TDOs post-CN^-^ and SCN^-^ exposure, respectively.
• The presented data can be utilized by researchers for comparative studies related to CN^-^ and SCN^-^ biodegradation.
• The bacterial organisms detected in both the CDOs and TDOs were mainly dominated by bacteria which have never been reported to possess CN^-^ and SCN^-^ degradation capabilities, and future research necessitates for the determination of the role that these organisms play in CN^-^ and SCN^-^ biodegradation processes.

## 1 Data

The presented dataset contains the bacterial composition of free cyanide (CDO) and thiocyanate degrading (TDO) organisms from electroplating and synthetic SCN^-^ containing wastewater, respectively. Table 1 shows the comparative analysis of the bacterial compositions between the CDOs and TDOs.

**Table 1:** Comparative analysis of the CDO and TDO bacterial communities.

**CDO**			**TDO**		
**Organism**	**% Abundance**	**Accession**	**Organism**	**% Abundance**	**Accession**
*Myroides odoratimimus*	35.26	gi|922317158|gb|KR349266.1|	*Myroides odoratimimus*	37.82	gi|163932218|gb|EU331413.1|
*Proteus sp.*	17.58	gi|189409506|gb|EU710747.1|	*Proteus vulgaris*	30.50	gi|923095386|gb|KP969052.1|
*Myroides sp.*	4.86	gi|914702437|gb|KP823024.1|	Uncultured bacterium	6.71	gi|648092936|gb|KJ604130.1|
*Stenotrophomonas maltophilia*	3.88	gi|194346582|gb|CP001111.1|	*Myroides sp.*	4.81	gi|736012191|gb|CP010327.1|
*Proteus mirabilis*	3.88	gi|333353439|gb|JF772095.1|	Uncultured proteus	2.54	gi|506969934|gb|KC896751.1|
Uncultured Enterobacteriaceae	3.86	gi|294613661|gb|GU905819.1|	*Stenotrophomonas maltophilia*	2.25	gi|194346582|gb|CP001111.1|
Uncultured *Proteus*	3.41	gi|506969934|gb|KC896751.1|	Uncultured providencia	1.54	gi|926458287|dbj|LC079061.1|
*Proteus vulgaris*	1.67	gi|340025986|gb|JN092605.1|	*Acidovorax sp.*	0.87	gi|120604516|gb|CP000539.1|
*Delftia sp.*	1.31	gi|333741867|gb|CP002735.1|	*Delftia sp.*	0.67	gi|333741867|gb|CP002735.1|
Uncultured *Thiobacillus*	1.26	gi|926657308|dbj|LC000812.1|	*Delftia acidovorans*	0.49	gi|160361034|gb|CP000884.1|
Uncultured *Providencia*	1.08	gi|926458287|dbj|LC079061.1|	*Pseudomonas syringae*	0.36	gi|63253978|gb|CP000075.1|
*Delftia acidovorans*	0.73	gi|160361034|gb|CP000884.1|	*Citrobacter koseri*	0.35	gi|673531252|emb|LK931336.1|
*Myroides profundi*	0.49	gi|753770668|gb|CP010817.1|	*Alicycliphilus denitrificans*	0.28	gi|329308025|gb|CP002657.1|
*Proteus penneri*	0.40	gi|919500502|gb|KT427910.1|	*Ralstonia solanacearum*	0.26	gi|916490054|gb|CP011997.1|
*Providencia vermicola*	0.39	gi|340026009|gb|JN092796.1|	Uncultured thiobacillus	0.25	gi|698322799|gb|KM595276.1|
*Klebsiella pneumoniae*	0.37	gi|926677775|gb|CP012300.1|	*Pseudomonas aeruginosa*	0.24	gi|660504631|gb|CP008749.1|
*Pseudomonas syringae*	0.37	gi|63253978|gb|CP000075.1|	*Sideroxydans lithotrophicus*	0.24	gi|291582584|gb|CP001965.1|
*Acidovorax sp.*	0.33	gi|407894523|gb|CP003872.1|	*Oceanimonas sp.*	0.24	gi|444439651|ref|NR_074966.1|
*Alcaligenes sp.*	0.28	gi|485951523|gb|KC534482.1|	*Serratia marcescens*	0.23	gi|560171871|emb|HG326223.1|
*Serratia marcescens*	0.24	gi|560171871|emb|HG326223.1|	Uncultured *Dokdonella*	0.23	gi|107785044|gb|DQ533520.1|
*Comamonas testosteroni*	0.22	gi|672605233|gb|CP006704.1|	*Providencia sp.*	0.22	gi|815932210|gb|KR232641.1|
*Ralstonia pickettii*	0.19	gi|546340292|gb|CP006668.1|	*Cupriavidus necator*	0.21	gi|338167938|gb|CP002878.1|
*Providencia sp.*	0.19	gi|815932210|gb|KR232641.1|	*Pseudomonas aeruginosa*	0.21	gi|915391195|dbj|AP014839.2|
*Cellulomonas flavigena*	0.16	gi|296019684|gb|CP001964.1|	*Pseudomonas chlororaphis*	0.19	gi|829490642|gb|CP011020.1|
*Pseudomonas putida*	0.14	gi|158392725|dbj|AB333783.1|	*Alicycliphilus denitrificans*	0.19	gi|329312633|gb|CP002658.1|

## 2 Experimental design, materials and methods

### 2.1 Sample collection and isolation procedure

The CDOs were isolated from an electroplating facility wastewater. The wastewater was collected in sterile non-transparent 20 L polypropylene containers and the cyanide concentration was immediately quantified to be above 150 mg CN^-^/L, using the detection technique developed by [1]. The TDOs were isolated from synthetic SCN^-^-containing wastewater solution (500 mL) containing (g/L); K_2_HPO_4_ (3.4), KH_2_PO_4_ (4.3), Glucose (0.01), SCN^-^ (0.2) and CN^-^ (0.2), at a pH of 10 (±0.05), using the gravimetric technique. Briefly, the solution was exposed for two months to allow airborne microorganisms to settle on the media outside the laboratory. A fraction (100 mL) of both the synthetic and electroplating wastewater solutions was filtered sterilized in a 0.22 µm Millipore membrane and the microbial cells were re-suspended in 5 mL of sterile Millipore water in preparation of DNA extraction procedures.

### 2.2 DNA extraction and Sequencing

The metacommunity DNA was extracted directly from the CDO and TDO re-suspension solutions, using commercially available extraction kits (Promega, Madison, Wisconsin, USA), as per manufacturer׳s instructions. The 16S rRNA forward bacterial primers 27F-16S-5′-AGAGTTTGATCMTGGCTCAG-′3 and reverse primers 518R-16S-5′-ATTACCGCGGCTGCTGG-′3 [2] that targeted the V1 and V3 regions of the 16S rRNA were used for the PCR amplification of the purified DNA samples. The PCR amplicons were gel purified, end repaired and illumina® specific adapter sequence were ligated to each amplicon. Following quantification and purification steps, the amplicons were then sequenced using the illumina® MiSeq-2000, using a MiSeq V3 (600 cycle) kit. 20 Mb of the data (2×300 bp long paired end reads) were produced for each sample as described previously [3]. The Basic Local Alignment Search Tool (BLAST)-based data analysis was performed with the assistance of an Inqaba Biotec (Pretoria, South Africa) in-house developed data analysis pipeline.

## Footnotes

Appendix A Supplementary data associated with this article can be found in the online version at http://dx.doi.org/10.1016/j.dib.2017.06.049.

## References

[1] Lambert J.L., Ramasamy J., Paukstelis J.V. (1975). Stable reagents for the colorimetric determination of cyanide by modified Koenig reactions. Anal. Chem..

[2] Satokari R.M., Vaughan E.E., Akkermans A.D., Saarela M., De Vos W.M. (2001). Bifidobacterial diversity in human feces detected by genus-specific PCR and denaturing gradient gel electrophoresis. Appl. Environ. Microbiol.

[3] Das S., Bora S.S., Yadav R.N.S., Barooah M. (2017). A metagenomic approach to decipher the indigenous microbial communities of arsenic contaminated groundwater of Assam. Genom. Data.

