# Supplementary material for: Metagenomic data of free cyanide and thiocyanate degrading bacterial communities
Source: Data Brief. 2017 Jul 4;13:738–41. doi: 10.1016/j.dib.2017.06.049 (PMC5512189; doi:10.1016/j.dib.2017.06.049)
Supplement: Supplementary file 2 — Supplementary material [file mmc2.docx]

**Table 1**: The bacterial community composition of the CDO as identified by 16S rDNA amplicon gene sequencing.

| Organism/HIT | Cluster size | % | Accession | e-value | Fastq header |
| --- | --- | --- | --- | --- | --- |
| *Myroides odoratimimus* | 3147 | 37.82 | gi\|163932218\|gb\|EU331413.1\| | 1.2932e-114 | M01232:1:000000000-AP50K:1:1104:14758:19235 |
| *Proteus vulgaris* | 2538 | 30.50 | gi\|923095386\|gb\|KP969052.1\| | 2.72887e-79 | M01232:1:000000000-AP50K:1:1103:23348:5397 |
| Uncultured bacterium | 558 | 6.71 | gi\|648092936\|gb\|KJ604130.1\| | 3.69942e-100 | M01232:1:000000000-AP50K:1:1104:13883:7954 |
| *Myroides sp.* | 400 | 4.81 | gi\|736012191\|gb\|CP010327.1\| | 5.99806e-93 | M01232:1:000000000-AP50K:1:1108:8233:2199 |
| Uncultured proteus | 211 | 2.54 | gi\|506969934\|gb\|KC896751.1\| | 1.62701e-93 | M01232:1:000000000-AP50K:1:2101:19182:18633 |
| *Stenotrophomonas maltophilia* | 187 | 2.25 | gi\|194346582\|gb\|CP001111.1\| | 1.46331e-98 | M01232:1:000000000-AP50K:1:1119:11670:13420 |
| Uncultured providencia | 128 | 1.54 | gi\|926458287\|dbj\|LC079061.1\| | 3.43811e-41 | M01232:1:000000000-AP50K:1:1104:5503:20191 |
| *Acidovorax sp.* | 72 | 0.87 | gi\|120604516\|gb\|CP000539.1\| | 1.3288e-33 | M01232:1:000000000-AP50K:1:1101:2376:13124 |
| *Delftia sp.* | 56 | 0.67 | gi\|333741867\|gb\|CP002735.1\| | 3.87294e-18 | M01232:1:000000000-AP50K:1:1108:16411:13013 |
| *Delftia acidovorans* | 41 | 0.49 | gi\|160361034\|gb\|CP000884.1\| | 3.47335e-57 | M01232:1:000000000-AP50K:1:1103:9363:7746 |
| *Pseudomonas syringae* | 30 | 0.36 | gi\|63253978\|gb\|CP000075.1\| | 2.99499e-63 | M01232:1:000000000-AP50K:1:1105:18596:23856 |
| *Citrobacter koseri* | 29 | 0.35 | gi\|673531252\|emb\|LK931336.1\| | 8.60737e-12 | M01232:1:000000000-AP50K:1:1104:4495:18351 |
| *Alicycliphilus denitrificans* | 23 | 0.28 | gi\|329308025\|gb\|CP002657.1\| | 3.05065e-16 | M01232:1:000000000-AP50K:1:1105:8530:19327 |
| *Ralstonia solanacearum* | 22 | 0.26 | gi\|916490054\|gb\|CP011997.1\| | 1.00598e-75 | M01232:1:000000000-AP50K:1:1108:24493:12339 |
| Uncultured thiobacillus | 21 | 0.25 | gi\|698322799\|gb\|KM595276.1\| | 5.50735e-148 | M01232:1:000000000-AP50K:1:1105:12282:9420 |
| *Pseudomonas aeruginosa* | 20 | 0.24 | gi\|660504631\|gb\|CP008749.1\| | 1.19213e-144 | M01232:1:000000000-AP50K:1:1103:9815:13566 |
| *Sideroxydans lithotrophicus* | 20 | 0.24 | gi\|291582584\|gb\|CP001965.1\| | 4.90792e-53 | M01232:1:000000000-AP50K:1:1108:11320:23576 |
| *Oceanimonas sp.* | 20 | 0.24 | gi\|444439651\|ref\|NR_074966.1\| | 7.35192e-27 | M01232:1:000000000-AP50K:1:1119:3722:11659 |
| *Serratia marcescens* | 19 | 0.23 | gi\|560171871\|emb\|HG326223.1\| | 1.85544e-49 | M01232:1:000000000-AP50K:1:2101:18939:17690 |
| *Comamonas testosteroni* | 18 | 0.22 | gi\|672605233\|gb\|CP006704.1\| | 3.59636e-90 | M01232:1:000000000-AP50K:1:1105:10705:11355 |
| *Ralstonia pickettii* | 16 | 0.19 | gi\|546340292\|gb\|CP006668.1\| | 1.82435e-08 | M01232:1:000000000-AP50K:1:2101:18026:5128 |
| *Providencia sp.* | 16 | 0.19 | gi\|815932210\|gb\|KR232641.1\| | 2.72887e-79 | M01232:1:000000000-AP50K:1:1103:25689:12753 |
| *Cellulomonas flavigena* | 13 | 0.16 | gi\|296019684\|gb\|CP001964.1\| | 2.62834e-31 | M01232:1:000000000-AP50K:1:1101:19393:21486 |
| *Pseudomonas putida* | 12 | 0.14 | gi\|158392725\|dbj\|AB333783.1\| | 2.2351e-49 | M01232:1:000000000-AP50K:1:1104:20333:19760 |
| *Acidovorax ebreus* | 11 | 0.13 | gi\|221728669\|gb\|CP001392.1\| | 4.52077e-89 | M01232:1:000000000-AP50K:1:2101:15985:18363 |
| *Pseudomonas chlororaphis* | 9 | 0.11 | gi\|787852299\|gb\|CP011110.1\| | 2.86247e-61 | M01232:1:000000000-AP50K:1:1101:11528:15699 |
| *Achromobacter xylosoxidans* | 9 | 0.11 | gi\|408362959\|gb\|JX448550.1\| | 7.23576e-132 | M01232:1:000000000-AP50K:1:1101:11140:19395 |
| *Klebsiella oxytoca* | 8 | 0.10 | gi\|828959694\|gb\|CP011636.1\| | 6.02932e-138 | M01232:1:000000000-AP50K:1:1119:15407:13718 |
| *Pseudoxanthomonas suwonensis* | 7 | 0.08 | gi\|317464132\|gb\|CP002446.1\| | 5.35019e-44 | M01232:1:000000000-AP50K:1:1103:12838:17520 |
| *Proteus sp.* | 7 | 0.08 | gi\|914702447\|gb\|KP823034.1\| | 1.22143e-144 | M01232:1:000000000-AP50K:1:1105:16106:13146 |
| *Myroides profundi* | 7 | 0.08 | gi\|753770668\|gb\|CP010817.1\| | 2.72036e-42 | M01232:1:000000000-AP50K:1:1119:24298:13050 |
| *Herbaspirillum seropedicae* | 7 | 0.08 | gi\|852454696\|gb\|CP011930.1\| | 9.9185e-86 | M01232:1:000000000-AP50K:1:1108:17746:12414 |
| *Alcaligenes sp.* | 7 | 0.08 | gi\|409103805\|dbj\|AB754812.1\| | 3.58594e-99 | M01232:1:000000000-AP50K:1:1103:11208:1154 |
| *Bacillus cereus* | 6 | 0.07 | gi\|755995789\|gb\|CP009605.1\| | 1.1257e-139 | M01232:1:000000000-AP50K:1:1105:14482:24175 |
| *Pseudomonas plecoglossicida* | 5 | 0.06 | gi\|752308899\|gb\|CP010359.1\| | 1.67789e-78 | M01232:1:000000000-AP50K:1:1108:15191:16124 |
| *Propionibacterium acnes* | 4 | 0.05 | gi\|657118275\|gb\|CP006032.1\| e | 2.54479e-136 | M01232:1:000000000-AP50K:1:1103:8162:17028 |
| *Azotobacter chroococcum* | 4 | 0.05 | gi\|747125374\|gb\|CP010415.1\| | 2.93158e-36 | M01232:1:000000000-AP50K:1:2101:13802:22643 |
| *Alcaligenes faecalis* | 4 | 0.05 | gi\|913125411\|gb\|KP859538.1\| | 1.81552e-77 | M01232:1:000000000-AP50K:1:1119:9072:15900 |
| *Cupriavidus necator* | 4 | 0.05 | gi\|338167938\|gb\|CP002878.1\| | 1.74718e-68 | M01232:1:000000000-AP50K:1:1105:6376:19499 |
| *Proteus mirabilis* | 3 | 0.04 | gi\|806981489\|gb\|KP401767.1\| | 5.52136e-34 | M01232:1:000000000-AP50K:1:1108:19368:22094 |
| *Pseudomonas protegens* | 3 | 0.04 | gi\|68342549\|gb\|CP000076.1\| | 1.24973e-39 | M01232:1:000000000-AP50K:1:1101:20194:24301 |
| *Enterobacter cloacae* | 3 | 0.04 | gi\|846395709\|gb\|KT157602.1\| | 1.00666e-19 | M01232:1:000000000-AP50K:1:1105:11563:20149 |
| *Xanthomonas sacchari* | 3 | 0.04 | gi\|743685209\|gb\|CP010409.1\| | 6.08919e-73 | M01232:1:000000000-AP50K:1:1101:20357:11233 |
| *Pseudomonas stutzeri* | 3 | 0.04 | gi\|390981275\|gb\|CP003677.1\| | 1.51862e-128 | M01232:1:000000000-AP50K:1:1103:11149:2076 |
| *Xanthomonas translucens* | 3 | 0.04 | gi\|828451590\|gb\|CP008714.1\| | 3.81639e-40 | M01232:1:000000000-AP50K:1:1108:10215:10753 |
| *Thiobacillus denitrificans* | 3 | 0.04 | gi\|74055513\|gb\|CP000116.1\| | 2.20356e-77 | M01232:1:000000000-AP50K:1:1119:12892:5872 |
| *Xanthomonas arboricola* | 3 | 0.04 | gi\|814589807\|gb\|KP314293.1\| | 1.64046e-22 | M01232:1:000000000-AP50K:1:1119:21701:4832 |
| *Escherichia coli* | 3 | 0.04 | gi\|926539378\|gb\|CP012632.1\| | 2.10591e-97 | M01232:1:000000000-AP50K:1:1108:9185:19967 |
| *Herbaspirillum frisingense* | 3 | 0.04 | gi\|896689380\|gb\|KP713806.1\| | 2.19511e-137 | M01232:1:000000000-AP50K:1:1108:19475:16710 |
| Uncultured dokdonella | 3 | 0.04 | gi\|107785044\|gb\|DQ533520.1\| | 5.97996e-81 | M01232:1:000000000-AP50K:1:1101:23990:19816 |
| Pigmentiphaga sp. | 3 | 0.04 | gi\|338319330\|gb\|JN000323.1\| | 5.06188e-133 | M01232:1:000000000-AP50K:1:1108:16603:23108 |
| *Pseudomonas alkylphenolia* | 3 | 0.04 | gi\|675318909\|gb\|CP009048.1\| | 8.16048e-47 | M01232:1:000000000-AP50K:1:1101:13291:10977 |
| *Thiobacillus sp*. | 2 | 0.02 | gi\|239836913\|gb\|FJ982929.1\| | 6.91686e-38 | M01232:1:000000000-AP50K:1:1103:22517:10320 |
| *Cupriavidus metallidurans* | 2 | 0.02 | gi\|93352797\|gb\|CP000352.1\| | 5.55178e-24 | M01232:1:000000000-AP50K:1:1108:22378:4923 |
| Uncultured stenotrophomonas | 2 | 0.02 | gi\|410699391\|gb\|JX575903.1\| | 1.22403e-124 | M01232:1:000000000-AP50K:1:1119:23419:5859 |
| *Pseudomonas entomophila* | 2 | 0.02 | gi\|95101722\|emb\|CT573326.1\| | 2.95291e-96 | M01232:1:000000000-AP50K:1:1103:16389:8246 |
| *Bordetella bronchiseptica* | 2 | 0.02 | gi\|408767172\|emb\|HE965806.1\| | 1.00913e-55 | M01232:1:000000000-AP50K:1:1103:26322:7711 |
| *Pseudomonas cremoricolorata* | 2 | 0.02 | gi\|691224436\|gb\|CP009455.1\| | 1.05031e-35 | M01232:1:000000000-AP50K:1:2101:21852:15501 |
| *Xanthobacter sp.* | 2 | 0.02 | gi\|399905791\|gb\|JX178938.1\| | 3.57787e-135 | M01232:1:000000000-AP50K:1:1101:22732:9041 |
| *Stenotrophomonas rhizophila* | 2 | 0.02 | gi\|627787876\|gb\|CP007597.1\| | 7.77009e-87 | M01232:1:000000000-AP50K:1:1103:13343:23439 |
| *Acidovorax avenae* | 2 | 0.02 | gi\|323371659\|gb\|CP002521.1\| | 2.01012e-62 | M01232:1:000000000-AP50K:1:1108:10203:16891 |
| *Pseudomonas sp.* | 2 | 0.02 | gi\|568237447\|gb\|CP007012.1\| | 2.40657e-72 | M01232:1:000000000-AP50K:1:1104:8727:20913 |
| *Dechloromonas aromatica* | 2 | 0.02 | gi\|71845263\|gb\|CP000089.1\| | 1.16024e-25 | M01232:1:000000000-AP50K:1:1101:11771:4495 |
| *Pseudomonas fluorescens* | 2 | 0.02 | gi\|800910178\|gb\|CP011117.1\| | 2.11681e-92 | M01232:1:000000000-AP50K:1:1101:27585:14039 |
| *Burkholderia glumae* | 2 | 0.02 | gi\|755901546\|gb\|CP002581.1\| | 1.80799e-13 | M01232:1:000000000-AP50K:1:1103:16164:15424 |
| *Anaplasma marginale* | 2 | 0.02 | gi\|5852413\|gb\|AF112479.1\| | 5.57527e-138 | M01232:1:000000000-AP50K:1:1119:25107:13616 |
| Uncultured glaciimonas | 1 | 0.01 | gi\|814556810\|gb\|KP794262.1\| | 5.44244e-108 | M01232:1:000000000-AP50K:1:1101:17921:23187 |
| *Gemmatirosa kalamazoonesis* | 1 | 0.01 | gi\|575461033\|gb\|CP007129.1\| | 1.58003e-18 | M01232:1:000000000-AP50K:1:1108:19813:13307 |
| *Streptomyces avermitilis* | 1 | 0.01 | gi\|148878541\|dbj\|BA000030.3\| | 4.88333e-44 | M01232:1:000000000-AP50K:1:1108:6176:5007 |
| *Dyella jiangningensis* | 1 | 0.01 | gi\|612502010\|gb\|CP007444.1\| | 6.607e-18 | M01232:1:000000000-AP50K:1:1119:12136:3897 |
| *Ramlibacter tataouinensis* | 1 | 0.01 | gi\|334728683\|gb\|CP000245.1\| | 1.78717e-28 | M01232:1:000000000-AP50K:1:1108:14678:6930 |
| *Xanthomonas campestris* | 1 | 0.01 | gi\|807234606\|gb\|CP011256.1\| | 1.26649e-74 | M01232:1:000000000-AP50K:1:1103:21055:2238 |
| *Burkholderia multivorans* | 1 | 0.01 | gi\|773019683\|gb\|CP009830.1\| | 8.70161e-37 | M01232:1:000000000-AP50K:1:1105:6870:12764 |
| *Pseudomonas resinovorans* | 1 | 0.01 | gi\|512374267\|dbj\|AP013068.1\| | 1.39069e-78 | M01232:1:000000000-AP50K:1:1108:4168:6698 |
| *Enterobacter asburiae* | 1 | 0.01 | gi\|918042828\|gb\|CP010360.2\| | 3.88117e-94 | M01232:1:000000000-AP50K:1:1104:12559:22562 |
| *Kurthia sp.* | 1 | 0.01 | gi\|512134489\|gb\|KF023517.1\| | 3.51958e-85 | M01232:1:000000000-AP50K:1:2101:21640:20880 |
| *Pseudomonas fulva* | 1 | 0.01 | gi\|333113473\|gb\|CP002727.1\| | 6.09224e-78 | M01232:1:000000000-AP50K:1:1105:13315:17367 |
| *Bradyrhizobium japonicum* | 1 | 0.01 | gi\|736032532\|gb\|CP010313.1\| | 3.56572e-45 | M01232:1:000000000-AP50K:1:1101:7505:23933 |
| *Modestobacter marinus* | 1 | 0.01 | gi\|388483940\|emb\|FO203431.1\| | 2.57433e-17 | M01232:1:000000000-AP50K:1:1119:11022:20109 |
| Uncultured aminobacter | 1 | 0.01 | gi\|507579830\|gb\|KC923256.1\| | 3.8135e-62 | M01232:1:000000000-AP50K:1:1103:20934:5907 |
| *Laribacter hongkongensis* | 1 | 0.01 | gi\|226713858\|gb\|CP001154.1\| | 1.13729e-20 | M01232:1:000000000-AP50K:1:1105:18399:14089 |
| *Pseudomonas mendocina* | 1 | 0.01 | gi\|145573243\|gb\|CP000680.1\| | 2.70299e-41 | M01232:1:000000000-AP50K:1:1108:5781:14023 |
| *Saccharothrix espanaensis* | 1 | 0.01 | gi\|407879691\|emb\|HE804045.1\| | 4.50833e-05 | M01232:1:000000000-AP50K:1:1105:17029:25180 |
| *Blastococcus saxobsidens* | 1 | 0.01 | gi\|378781357\|emb\|FO117623.1\| | 2.3524e-77 | M01232:1:000000000-AP50K:1:1108:20155:17758 |
| *Arsenophonus endosymbiont* | 1 | 0.01 | gi\|820660972\|emb\|LN829878.1\| | 1.32302e-09 | M01232:1:000000000-AP50K:1:1119:20738:5938 |
| *Nocardioides sp.* | 1 | 0.01 | gi\|119534933\|gb\|CP000509.1\| | 3.01954e-11 | M01232:1:000000000-AP50K:1:1105:19224:4938 |
| *Methylovorus sp.* | 1 | 0.01 | gi\|312439093\|gb\|CP002252.1\| | 2.78443e-36 | M01232:1:000000000-AP50K:1:1105:24289:17306 |
| *Clostridium sp*. | 1 | 0.01 | gi\|923889233\|gb\|KM083029.1\| | 3.44947e-30 | M01232:1:000000000-AP50K:1:1119:12173:7246 |
| *Dechlorosoma suillum* | 1 | 0.01 | gi\|359353254\|gb\|CP003153.1\| | 1.47032e-108 | M01232:1:000000000-AP50K:1:1105:4375:9732 |
| *Burkholderia cepacia* | 1 | 0.01 | gi\|685655815\|gb\|CP007785.1\| | 9.83606e-06 | M01232:1:000000000-AP50K:1:1119:16950:13814 |
| *Pseudomonadaceae bacterium* | 1 | 0.01 | gi\|915642179\|gb\|CP012365.1\| | 4.27957e-144 | M01232:1:000000000-AP50K:1:1119:14734:22296 |
| *Pseudomonas trivialis* | 1 | 0.01 | gi\|902687210\|gb\|CP011507.1\| | 3.75952e-100 | M01232:1:000000000-AP50K:1:1101:15256:17020 |
| *Xanthomonas citri* | 1 | 0.01 | gi\|780460238\|gb\|CP009039.1\| | 6.18065e-118 | M01232:1:000000000-AP50K:1:1108:12086:4164 |
| Uncultured propionibacteriaceae | 1 | 0.01 | gi\|545345141\|gb\|KF508373.1\| | 5.93388e-133 | M01232:1:000000000-AP50K:1:1105:6659:18480 |
| *Caulobacter segnis* | 1 | 0.01 | gi\|295429362\|gb\|CP002008.1\| | 1.68138e-36 | M01232:1:000000000-AP50K:1:1101:11506:18029 |
| *Pandoraea thiooxydans* | 1 | 0.01 | gi\|827097060\|gb\|CP011568.1\| | 2.40553e-11 | M01232:1:000000000-AP50K:1:2101:25977:8720 |
| *Tenacibaculum sp.* | 1 | 0.01 | gi\|923091939\|gb\|KT276410.1\| | 1.37719e-17 | M01232:1:000000000-AP50K:1:2101:18458:11220 |
| *Parvibaculum lavamentivorans* | 1 | 0.01 | gi\|154154406\|gb\|CP000774.1\| | 2.29925e-29 | M01232:1:000000000-AP50K:1:1101:14565:4126 |
| Uncultured ruminobacillus | 1 | 0.01 | gi\|388890795\|gb\|JQ724340.1\| | 8.80841e-131 | M01232:1:000000000-AP50K:1:1105:8600:23285 |
| *Craurococcus sp.* | 1 | 0.01 | gi\|578897545\|gb\|KF309173.1\| | 5.93388e-133 | M01232:1:000000000-AP50K:1:1105:6458:11514 |
| *Corallococcus coralloides* | 1 | 0.01 | gi\|380727201\|gb\|CP003389.1\| | 8.91475e-07 | M01232:1:000000000-AP50K:1:1104:14625:10752 |
| *Acidovorax citrulli* | 1 | 0.01 | gi\|120587178\|gb\|CP000512.1\| | 4.23107e-44 | M01232:1:000000000-AP50K:1:1105:23289:17689 |
| *Herbaspirillum sp.* | 1 | 0.01 | gi\|834963733\|gb\|KR296697.1\| | 5.27165e-88 | M01232:1:000000000-AP50K:1:1104:5122:11962 |
| *Devosia sp.* | 1 | 0.01 | gi\|901895891\|gb\|CP011300.1\| | 1.39026e-34 | M01232:1:000000000-AP50K:1:1119:9483:6762 |
| *Bacillus gaemokensis* | 1 | 0.01 | gi\|926657055\|dbj\|LC076295.1\| | 1.22202e-40 | M01232:1:000000000-AP50K:1:1104:15201:19946 |
| *Acidiphilium multivorum* | 1 | 0.01 | gi\|325049009\|dbj\|AP012035.1\| | 6.12555e-23 | M01232:1:000000000-AP50K:1:1101:15315:23043 |
| *Streptosporangium roseum* | 1 | 0.01 | gi\|270504784\|gb\|CP001814.1\| | 5.4811e-08 | M01232:1:000000000-AP50K:1:2101:15995:20274 |
| *Ricinus communis* | 1 | 0.01 | gi\|255603892\|ref\|XM_002538083.1\| | 2.11948e-81 | M01232:1:000000000-AP50K:1:1101:25137:19348 |
| *Arthrobacter sp*. | 1 | 0.01 | gi\|222546856\|gb\|FJ610336.1\| | 6.81216e-25 | M01232:1:000000000-AP50K:1:1105:17040:4664 |
| Uncultured acidovorax | 1 | 0.01 | gi\|922671874\|gb\|KP967502.1\| | 6.91686e-38 | M01232:1:000000000-AP50K:1:1105:9255:21314 |
| Uncultured ochrobactrum | 1 | 0.01 | gi\|926657571\|dbj\|LC001074.1\| | 1.67328e-81 | M01232:1:000000000-AP50K:1:1119:10734:15760 |
| *Mesorhizobium ciceri* | 1 | 0.01 | gi\|317165637\|gb\|CP002447.1\| | 1.08237e-30 | M01232:1:000000000-AP50K:1:1101:6481:9079 |
| *Azoarcus aromaticum* | 1 | 0.01 | gi\|56311475\|emb\|CR555306.1\| | 2.75857e-111 | M01232:1:000000000-AP50K:1:1104:17293:11038 |
| *Pseudomonas mosselii* | 1 | 0.01 | gi\|684194542\|gb\|CP009365.1\| | 1.04619e-50 | M01232:1:000000000-AP50K:1:1108:10764:6978 |
| *Mycobacterium rhodesiae* | 1 | 0.01 | gi\|359817839\|gb\|CP003169.1\| | 6.88467e-43 | M01232:1:000000000-AP50K:1:1119:26019:17592 |
| *Janthinobacterium Agaricidamnosum* | 1 | 0.01 | gi\|571265423\|emb\|HG322949.1\| | 1.91386e-23 | M01232:1:000000000-AP50K:1:1104:6020:12652 |
| *Variovorax paradoxus* | 1 | 0.01 | gi\|239799596\|gb\|CP001635.1\| | 9.36593e-11 | M01232:1:000000000-AP50K:1:1108:22494:17203 |
| *Micrococcus luteus* | 1 | 0.01 | gi\|758182997\|gb\|CP007437.1\| | 8.50581e-72 | M01232:1:000000000-AP50K:1:1101:7524:5746 |
| *Desulfobacterium Autotrophicum* | 1 | 0.01 | gi\|223689840\|gb\|CP001087.1\| | 6.61231e-08 | M01232:1:000000000-AP50K:1:1108:10710:15747 |

**Table 2**: The bacterial community composition of the TDO as identified by 16S rDNA amplicon gene sequencing.

| **Organism/HIT** | **Cluster size** | **%** | **Accession** | **e-value** | **Fastq header** | |
| --- | --- | --- | --- | --- | --- | --- |
| *Myroides odoratimimus* | 4847 | 35.26 | gi\|922317158\|gb\|KR349266.1\| | 3.77534e-46 | M01232:1:000000000-AP50K:1:1103:13905:16356 | |
| *Proteus sp.* | 2417 | 17.58 | gi\|189409506\|gb\|EU710747.1\| | 2.00902e-137 | M01232:1:000000000-AP50K:1:1108:12924:3193 | |
| *Myroides sp.* | 668 | 4.86 | gi\|914702437\|gb\|KP823024.1\| | 1.37719e-17 | M01232:1:000000000-AP50K:1:1103:24983:10053 | |
| *Stenotrophomonas maltophilia* | 534 | 3.88 | gi\|194346582\|gb\|CP001111.1\| | 5.57527e-138 | M01232:1:000000000-AP50K:1:1119:19648:16035 | |
| *Proteus mirabilis* | 533 | 3.88 | gi\|333353439\|gb\|JF772095.1\| | 9.68362e-111 | M01232:1:000000000-AP50K:1:1119:24016:17977 | |
| Uncultured Enterobacteriaceae | 530 | 3.86 | gi\|294613661\|gb\|GU905819.1\| | 2.62951e-106 | M01232:1:000000000-AP50K:1:2101:25386:13705 | |
| Uncultured *Proteus* | 469 | 3.41 | gi\|506969934\|gb\|KC896751.1\| | 1.21527e-99 | M01232:1:000000000-AP50K:1:1104:7992:2893 | |
| *Proteus vulgaris* | 229 | 1.67 | gi\|340025986\|gb\|JN092605.1\| | 1.24072e-129 | M01232:1:000000000-AP50K:1:1108:28974:16380 |  |
| *Delftia sp.* | 180 | 1.31 | gi\|333741867\|gb\|CP002735.1\| | 2.35216e-48 | M01232:1:000000000-AP50K:1:2101:23435:5502 | |
| Uncultured *Thiobacillus* | 173 | 1.26 | gi\|926657308\|dbj\|LC000812.1\| | 3.38393e-125 | M01232:1:000000000-AP50K:1:1108:9976:11963 | |
| Uncultured *Providencia* | 149 | 1.08 | gi\|926458287\|dbj\|LC079061.1\| | 4.01632e-46 | M01232:1:000000000-AP50K:1:1119:28844:12035 | |
| *Delftia acidovorans* | 101 | 0.73 | gi\|160361034\|gb\|CP000884.1\| | 5.87347e-107 | M01232:1:000000000-AP50K:1:1108:20088:23867 | |
| *Myroides profundi* | 67 | 0.49 | gi\|753770668\|gb\|CP010817.1\| | 9.73837e-31 | M01232:1:000000000-AP50K:1:1105:18480:8337 | |
| *Proteus penneri* | 55 | 0.40 | gi\|919500502\|gb\|KT427910.1\| | 2.37446e-81 | M01232:1:000000000-AP50K:1:1104:11655:2456 | |
| *Providencia vermicola* | 54 | 0.39 | gi\|340026009\|gb\|JN092796.1\| | 1.56114e-133 | M01232:1:000000000-AP50K:1:1103:8136:16885 | |
| *Klebsiella pneumoniae* | 51 | 0.37 | gi\|926677775\|gb\|CP012300.1\| | 7.18337e-21 | M01232:1:000000000-AP50K:1:1105:23300:6043 | |
| *Pseudomonas syringae* | 51 | 0.37 | gi\|63253978\|gb\|CP000075.1\| | 4.73653e-61 | M01232:1:000000000-AP50K:1:1104:7000:18283 | |
| *Acidovorax sp.* | 46 | 0.33 | gi\|407894523\|gb\|CP003872.1\| | 3.9268e-14 | M01232:1:000000000-AP50K:1:1105:7372:15086 | |
| *Alcaligenes sp.* | 38 | 0.28 | gi\|485951523\|gb\|KC534482.1\| | 1.27439e-114 | M01232:1:000000000-AP50K:1:1104:13743:9344 | |
| *Serratia marcescens* | 33 | 0.24 | gi\|560171871\|emb\|HG326223.1\| | 1.16284e-144 | M01232:1:000000000-AP50K:1:1103:6353:17408 | |
| Uncultured *Dokdonella* | 31 | 0.23 | gi\|107785044\|gb\|DQ533520.1\| | 4.25281e-139 | M01232:1:000000000-AP50K:1:1108:25742:19359 | |
| *Providencia sp.* | 30 | 0.22 | gi\|815932210\|gb\|KR232641.1\| | 6.01749e-148 | M01232:1:000000000-AP50K:1:1108:21355:17715 | |
| *Cupriavidus necator* | 29 | 0.21 | gi\|338167938\|gb\|CP002878.1\| | 7.25413e-67 | M01232:1:000000000-AP50K:1:1104:18041:23936 | |
| *Pseudomonas aeruginosa* | 29 | 0.21 | gi\|915391195\|dbj\|AP014839.2\| | 6.17238e-33 | M01232:1:000000000-AP50K:1:1119:13684:24016 | |
| *Pseudomonas chlororaphis* | 26 | 0.19 | gi\|829490642\|gb\|CP011020.1\| | 3.07976e-16 | M01232:1:000000000-AP50K:1:1119:25016:6318 | |
| *Alicycliphilus denitrificans* | 26 | 0.19 | gi\|329312633\|gb\|CP002658.1\| | 6.51491e-81 | M01232:1:000000000-AP50K:1:1119:16258:20862 | |
| *Thiobacillus sp.* | 21 | 0.15 | gi\|239836913\|gb\|FJ982929.1\| | 7.38599e-54 | M01232:1:000000000-AP50K:1:1101:12930:7768 | |
| *Achromobacter xylosoxidans* | 21 | 0.15 | gi\|777196577\|emb\|LN831029.1\| | 4.88815e-84 | M01232:1:000000000-AP50K:1:1105:21838:15968 | |
| *Microbacteriaceae bacterium* | 19 | 0.14 | gi\|601039660\|gb\|KJ023347.1\| | 1.24862e-149 | M01232:1:000000000-AP50K:1:1105:23690:13978 | |
| *Ralstonia solanacearum* | 19 | 0.14 | gi\|469772332\|gb\|CP004012.1\| | 1.12762e-65 | M01232:1:000000000-AP50K:1:1119:10340:2237 | |
| *Variovorax paradoxus* | 19 | 0.14 | gi\|239799596\|gb\|CP001635.1\| | 1.00844e-80 | M01232:1:000000000-AP50K:1:1119:19727:9107 | |
| *Cellulomonas flavigena* | 17 | 0.12 | gi\|296019684\|gb\|CP001964.1\| | 2.55423e-31 | M01232:1:000000000-AP50K:1:1105:13783:6005 | |
| Uncultured *Stenotrophomonas* | 16 | 0.12 | gi\|821346181\|dbj\|LC053309.1\| | 2.52978e-20 | M01232:1:000000000-AP50K:1:1104:24756:18070 | |
| *Providencia stuartii* | 14 | 0.10 | gi\|684200017\|gb\|CP008920.1\| | 4.41388e-56 | M01232:1:000000000-AP50K:1:1103:6346:13968 | |
| Uncultured *Pseudomonas* | 13 | 0.09 | gi\|923142338\|emb\|LN875100.1\| | 6.06021e-143 | M01232:1:000000000-AP50K:1:1104:6710:18297 | |
| *Acidovorax ebreus* | 12 | 0.09 | gi\|221728669\|gb\|CP001392.1\| | 1.58887e-13 | M01232:1:000000000-AP50K:1:1103:25280:18609 | |
| *Comamonas testosteroni* | 12 | 0.09 | gi\|672605233\|gb\|CP006704.1\| | 2.66179e-29 | M01232:1:000000000-AP50K:1:1103:26782:15303 | |
| *Herbaspirillum seropedicae* | 10 | 0.07 | gi\|852454696\|gb\|CP011930.1\| | 1.32919e-44 | M01232:1:000000000-AP50K:1:1104:21074:20151 | |
| *Sideroxydans lithotrophicus* | 10 | 0.07 | gi\|291582584\|gb\|CP001965.1\| | 6.09744e-68 | M01232:1:000000000-AP50K:1:1108:14355:17157 | |
| *Pseudomonas putida* | 9 | 0.07 | gi\|764072275\|gb\|CP010979.1\| | 4.70475e-89 | M01232:1:000000000-AP50K:1:1104:7851:7801 | |
| *Citrobacter koseri* | 9 | 0.07 | gi\|673531252\|emb\|LK931336.1\| | 8.34654e-12 | M01232:1:000000000-AP50K:1:1105:8269:13992 | |
| *Orientia tsutsugamushi* | 9 | 0.07 | gi\|33320065\|gb\|AF478127.1\| | 3.37748e-125 | M01232:1:000000000-AP50K:1:1103:7270:11422 | |
| *Pseudomonas stutzeri* | 8 | 0.06 | gi\|390981275\|gb\|CP003677.1\| | 2.59749e-131 | M01232:1:000000000-AP50K:1:1105:21094:18564 | |
| *Uncultured acidovorax* | 8 | 0.06 | gi\|926655361\|dbj\|LC001616.1\| | 3.40326e-125 | M01232:1:000000000-AP50K:1:1101:18683:14419 | |
| Uncultured *Hydrogenophilaceae* | 8 | 0.06 | gi\|821254099\|gb\|KP292554.1\| | 2.57936e-48 | M01232:1:000000000-AP50K:1:1101:9948:3857 | |
| *Bacillus sp.* | 7 | 0.05 | gi\|925176648\|gb\|KR006321.1\| | 6.49292e-132 | M01232:1:000000000-AP50K:1:1104:12716:23643 | |
| *Herbaspirillum frisingense* | 7 | 0.05 | gi\|378404942\|gb\|JN869241.1\| | 8.52369e-131 | M01232:1:000000000-AP50K:1:1104:12346:23855 | |
| Uncultured *Klebsiella* | 7 | 0.05 | gi\|926657574\|dbj\|LC001077.1\| | 1.37719e-17 | M01232:1:000000000-AP50K:1:1119:12742:4053 | |
| *Xanthomonas sacchari* | 5 | 0.04 | gi\|743685209\|gb\|CP010409.1\| | 2.22908e-82 | M01232:1:000000000-AP50K:1:1104:9161:3819 | |
| *Parastrongyloides trichosuri* | 5 | 0.04 | gi\|687022128\|emb\|LM523277.1\| | 2.07111e-102 | M01232:1:000000000-AP50K:1:1108:17853:10783 | |
| *Klebsiella oxytoca* | 4 | 0.03 | gi\|828959694\|gb\|CP011636.1\| | 3.41767e-130 | M01232:1:000000000-AP50K:1:1104:21217:13341 | |
| *Aestuariibaculum scopimerae* | 4 | 0.03 | gi\|926663105\|ref\|NR_132699.1\| | 4.83672e-17 | M01232:1:000000000-AP50K:1:1108:8816:18593 | |
| *Uncultured vibrionaceae* | 4 | 0.03 | gi\|588293873\|gb\|KF941760.1\| | 2.03886e-21 | M01232:1:000000000-AP50K:1:1119:12113:16557 | |
| *Burkholderia multivorans* | 4 | 0.03 | gi\|773019683\|gb\|CP009830.1\| | 9.03205e-37 | M01232:1:000000000-AP50K:1:1103:14186:4658 | |
| Uncultured *Alcaligenes* | 4 | 0.03 | gi\|364515557\|gb\|JN860163.1\| | 3.72554e-125 | M01232:1:000000000-AP50K:1:1104:14089:9091 | |
| *Laribacter hongkongensis* | 4 | 0.03 | gi\|226713858\|gb\|CP001154.1\| | 8.90157e-21 | M01232:1:000000000-AP50K:1:1119:16383:3337 | |
| *Clavibacter michiganensis* | 4 | 0.03 | gi\|472820487\|emb\|HE614873.1\| | 7.86571e-47 | M01232:1:000000000-AP50K:1:1104:5972:8945 | |
| *Pseudomonas sp.* | 4 | 0.03 | gi\|808352541\|gb\|KP289281.1\| | 9.36474e-136 | M01232:1:000000000-AP50K:1:1105:18131:13296 | |
| *Pseudomonas fluorescens* | 4 | 0.03 | gi\|666084728\|gb\|CP008896.1\| | 2.60607e-49 | M01232:1:000000000-AP50K:1:1103:5811:7452 | |
| *Agrobacterium rhizogenes* | 4 | 0.03 | gi\|300669338\|dbj\|AB289616.1\| | 3.33337e-145 | M01232:1:000000000-AP50K:1:1103:10729:19778 | |
| *Pseudomonas plecoglossicida* | 3 | 0.02 | gi\|752308899\|gb\|CP010359.1\| | 1.53648e-68 | M01232:1:000000000-AP50K:1:1104:20145:24300 | |
| *Streptomyces sp.* | 3 | 0.02 | gi\|822591927\|gb\|CP011492.1\| | 0.00174135 | M01232:1:000000000-AP50K:1:1119:18029:23483 | |
| *Spongitalea numazuensis* | 3 | 0.02 | gi\|926663071\|ref\|NR_132665.1\| | 4.83672e-17 | M01232:1:000000000-AP50K:1:1103:10377:6756 | |
| *Escherichia coli* | 3 | 0.02 | gi\|926539378\|gb\|CP012632.1\| | 1.03027e-95 | M01232:1:000000000-AP50K:1:1105:23506:18275 | |
| *Ralstonia pickettii* | 3 | 0.02 | gi\|187724002\|gb\|CP001068.1\| | 9.98039e-91 | M01232:1:000000000-AP50K:1:1103:24365:18270 | |
| Uncultured *Phaselicystis* | 3 | 0.02 | gi\|304422147\|gb\|HQ018497.1\| | 4.25525e-144 | M01232:1:000000000-AP50K:1:1108:11011:14887 | |
| *Xanthobacter sp.* | 3 | 0.02 | gi\|399905791\|gb\|JX178938.1\| | 4.52218e-139 | M01232:1:000000000-AP50K:1:1104:10445:20234 | |
| *Stenotrophomonas rhizophila* | 3 | 0.02 | gi\|627787876\|gb\|CP007597.1\| | 2.30476e-16 | M01232:1:000000000-AP50K:1:1108:16612:24154 | |
| Uncultured *Proteobacterium* | 3 | 0.02 | gi\|110754486\|gb\|DQ829173.1\| | 4.81929e-124 | M01232:1:000000000-AP50K:1:1104:9072:2651 | |
| *Salinicola zeshunii* | 3 | 0.02 | gi\|926663123\|ref\|NR_132717.1\| | 1.91635e-27 | M01232:1:000000000-AP50K:1:1104:21540:24452 | |
| *Pseudomonas alkylphenolia* | 3 | 0.02 | gi\|675318909\|gb\|CP009048.1\| | 1.04277e-55 | M01232:1:000000000-AP50K:1:1119:18207:15716 | |
| *Aminobacter sp.* | 3 | 0.02 | gi\|808352622\|gb\|KP792998.1\| | 2.92452e-63 | M01232:1:000000000-AP50K:1:1101:14457:21845 | |
| *Rhodopseudomonas palustris* | 2 | 0.01 | gi\|192282182\|gb\|CP001096.1\| | 3.24307e-31 | M01232:1:000000000-AP50K:1:1101:10107:11214 | |
| *Ramlibacter tataouinensis* | 2 | 0.01 | gi\|334728683\|gb\|CP000245.1\| | 6.456e-33 | M01232:1:000000000-AP50K:1:1101:14558:7299 | |
| *Pseudoxanthomonas suwonensis* | 2 | 0.01 | gi\|807383831\|gb\|CP011144.1\| | 3.79229e-08 | M01232:1:000000000-AP50K:1:1105:7165:13578 | |
| *Sulfuritalea hydrogenivorans* | 2 | 0.01 | gi\|572099409\|dbj\|AP012547.1\| | 1.29547e-34 | M01232:1:000000000-AP50K:1:1119:15635:22920 | |
| *Enterobacter asburiae* | 2 | 0.01 | gi\|918042828\|gb\|CP010360.2\| | 1.54314e-39 | M01232:1:000000000-AP50K:1:1101:11586:14538 | |
| *Tistrella mobilis* | 2 | 0.01 | gi\|388531416\|gb\|CP003239.1\| | 6.19294e-06 | M01232:1:000000000-AP50K:1:1119:19058:23555 | |
| *Pseudoxanthomonas Jiangsuensis* | 2 | 0.01 | gi\|926663118\|ref\|NR_132712.1\| | 2.72036e-42 | M01232:1:000000000-AP50K:1:1108:18564:16578 | |
| *Bacillus cereus* | 2 | 0.01 | gi\|925175099\|gb\|KP998178.1\| | 6.6322e-117 | M01232:1:000000000-AP50K:1:1104:14071:8360 | |
| *Pigmentiphaga sp.* | 2 | 0.01 | gi\|338319330\|gb\|JN000323.1\| | 4.68187e-82 | M01232:1:000000000-AP50K:1:1101:17204:19117 | |
| *Pseudomonas entomophila* | 2 | 0.01 | gi\|95101722\|emb\|CT573326.1\| | 6.66362e-77 | M01232:1:000000000-AP50K:1:1108:13845:5976 | |
| *Aeromonas salmonicida* | 2 | 0.01 | gi\|142849896\|gb\|CP000644.1\| | 8.28939e-16 | M01232:1:000000000-AP50K:1:1119:25813:20025 | |
| *Serratia sp.* | 2 | 0.01 | gi\|676307183\|gb\|CP003424.1\| | 4.6514e-56 | M01232:1:000000000-AP50K:1:1105:21737:8086 | |
| *Marinobacter similis* | 1 | 0.01 | gi\|582024311\|gb\|CP007151.1\| | 2.29529e-17 | M01232:1:000000000-AP50K:1:1103:13306:7456 | |
| Uncultured *Spirochaeta* | 1 | 0.01 | gi\|294821864\|gb\|HM049865.1\| | 1.29462e-79 | M01232:1:000000000-AP50K:1:1108:15441:22338 | |
| *Actinoplanes missouriensis* | 1 | 0.01 | gi\|381368402\|dbj\|AP012319.1\| | 2.85154e-61 | M01232:1:000000000-AP50K:1:1108:7406:8793 | |
| Uncultured *Actinobacterium* | 1 | 0.01 | gi\|146429963\|gb\|EF220779.1\| | 5.52247e-138 | M01232:1:000000000-AP50K:1:1108:15175:19488 | |
| *Brevundimonas sp.* | 1 | 0.01 | gi\|143024276\|gb\|EF486314.1\| | 5.65974e-138 | M01232:1:000000000-AP50K:1:1105:19075:3508 | |
| *Brevundimonas subvibrioides* | 1 | 0.01 | gi\|302191744\|gb\|CP002102.1\| | 2.9103e-56 | M01232:1:000000000-AP50K:1:1119:20428:17355 | |
| *Sphingomonas asaccharolytica* | 1 | 0.01 | gi\|780749594\|gb\|KP191993.1\| | 7.18337e-21 | M01232:1:000000000-AP50K:1:1101:7781:20498 | |
| *Salinicoccus iranensis* | 1 | 0.01 | gi\|343198876\|ref\|NR_043937.1\| | 0.00068987 | M01232:1:000000000-AP50K:1:1104:17030:4255 | |
| *Azoarcus sp.* | 1 | 0.01 | gi\|119668705\|emb\|AM406670.1\| | 1.4117e-06 | M01232:1:000000000-AP50K:1:1119:8538:22070 | |
| *Cronobacter muytjensii* | 1 | 0.01 | gi\|924333052\|gb\|CP012268.1\| | 6.91686e-38 | M01232:1:000000000-AP50K:1:1108:20035:10625 | |
| *Acidiphilium sp.* | 1 | 0.01 | gi\|908660394\|emb\|LN866594.1\| | 2.31342e-59 | M01232:1:000000000-AP50K:1:1104:13914:10793 | |
| *Delftia tsuruhatensis* | 1 | 0.01 | gi\|149774731\|gb\|EF469602.1\| | 8.57296e-121 | M01232:1:000000000-AP50K:1:1104:11900:8800 | |
| *Xanthomonas campestris* | 1 | 0.01 | gi\|902821146\|gb\|CP012145.1\| | 9.16951e-51 | M01232:1:000000000-AP50K:1:1101:3105:16542 | |
| *Kineococcus radiotolerans* | 1 | 0.01 | gi\|196121877\|gb\|CP000750.2\| | 4.17197e-88 | M01232:1:000000000-AP50K:1:2101:21228:23567 | |
| *Pusillimonas sp.* | 1 | 0.01 | gi\|687199282\|gb\|KM054873.1\| | 4.61262e-83 | M01232:1:000000000-AP50K:1:1101:5549:4857 | |
| *Halomonas campaniensis* | 1 | 0.01 | gi\|641739997\|gb\|CP007757.1\| | 1.72049e-88 | M01232:1:000000000-AP50K:1:1104:14866:17080 | |
| *Pseudomonas fulva* | 1 | 0.01 | gi\|333113473\|gb\|CP002727.1\| | 2.62623e-51 | M01232:1:000000000-AP50K:1:1103:11079:11827 | |
| *Bradyrhizobium japonicum* | 1 | 0.01 | gi\|736032532\|gb\|CP010313.1\| | 9.4916e-05 | M01232:1:000000000-AP50K:1:1119:2017:13951 | |
| *Leptothrix cholodnii* | 1 | 0.01 | gi\|170774137\|gb\|CP001013.1\| | 1.99438e-17 | M01232:1:000000000-AP50K:1:1101:12071:11566 | |
| *Cupriavidus metallidurans* | 1 | 0.01 | gi\|93352797\|gb\|CP000352.1\| | 1.77221e-38 | M01232:1:000000000-AP50K:1:1108:13285:6448 | |
| *Pseudomonas mendocina* | 1 | 0.01 | gi\|145573243\|gb\|CP000680.1\| | 7.95873e-62 | M01232:1:000000000-AP50K:1:1105:18267:8397 | |
| *Xanthomonas oryzae* | 1 | 0.01 | gi\|856757157\|gb\|CP011961.1\| | 6.73954e-63 | M01232:1:000000000-AP50K:1:1103:15563:18137 | |
| *Nocardioides sp.* | 1 | 0.01 | gi\|119534933\|gb\|CP000509.1\| | 1.15819e-35 | M01232:1:000000000-AP50K:1:1101:12688:21346 | |
| *Mycobacterium chelonae* | 1 | 0.01 | gi\|807052974\|gb\|CP010946.1\| | 1.62957e-28 | M01232:1:000000000-AP50K:1:1101:15655:1760 | |
| *Clostridium sp.* | 1 | 0.01 | gi\|723265869\|gb\|KM454168.1\| | 9.47417e-141 | M01232:1:000000000-AP50K:1:1104:12563:11135 | |
| *Burkholderia gladioli* | 1 | 0.01 | gi\|772900580\|gb\|CP009322.1\| | 1.10349e-15 | M01232:1:000000000-AP50K:1:1119:23014:19597 | |
| *Thiobacillus denitrificans* | 1 | 0.01 | gi\|74055513\|gb\|CP000116.1\| | 1.18486e-30 | M01232:1:000000000-AP50K:1:1104:15545:11426 | |
| *Alcaligenes faecalis* | 1 | 0.01 | gi\|913125411\|gb\|KP859538.1\| | 1.17868e-72 | M01232:1:000000000-AP50K:1:1119:16844:9553 | |
| Uncultured *Vampirovibrio* | 1 | 0.01 | gi\|765567848\|gb\|KP108877.1\| | 1.72989e-55 | M01232:1:000000000-AP50K:1:1105:22928:21695 | |
| *Rubrivivax gelatinosus* | 1 | 0.01 | gi\|381376528\|dbj\|AP012320.1\| | 1.09005e-25 | M01232:1:000000000-AP50K:1:1119:12241:9263 | |
| *Citrobacter freundii* | 1 | 0.01 | gi\|828983113\|gb\|CP011657.1\| | 2.9948e-31 | M01232:1:000000000-AP50K:1:1108:18565:17737 | |
| *Geobacillus sp.* | 1 | 0.01 | gi\|909995672\|gb\|CP008903.1\| | 6.13154e-22 | M01232:1:000000000-AP50K:1:1103:21864:18986 | |
| *Herbaspirillum hiltneri* | 1 | 0.01 | gi\|917675518\|gb\|CP011409.1\| | 1.82059e-23 | M01232:1:000000000-AP50K:1:1101:21629:10741 | |
| *Caulobacter segnis* | 1 | 0.01 | gi\|295429362\|gb\|CP002008.1\| | 4.918e-39 | M01232:1:000000000-AP50K:1:1105:9409:21765 | |
| *Pimelobacter simplex* | 1 | 0.01 | gi\|723622094\|gb\|CP009896.1\| | 1.10078e-10 | M01232:1:000000000-AP50K:1:1108:6955:12298 | |
| *Xanthomonas arboricola* | 1 | 0.01 | gi\|910827970\|gb\|CP012251.1\| | 3.01283e-32 | M01232:1:000000000-AP50K:1:1105:22121:13711 | |
| *Pseudomonas cremoricolorata* | 1 | 0.01 | gi\|691224436\|gb\|CP009455.1\| | 9.24404e-36 | M01232:1:000000000-AP50K:1:1105:17844:3878 | |
| *Bifidobacterium longum* | 1 | 0.01 | gi\|320456974\|dbj\|AP010889.1\| | 1.93619e-87 | M01232:1:000000000-AP50K:1:1108:25682:17588 | |
| *Caulobacter crescentus* | 1 | 0.01 | gi\|220962111\|gb\|CP001340.1\| | 2.94987e-61 | M01232:1:000000000-AP50K:1:1104:9818:9158 | |
| *Acidithiobacillus caldus* | 1 | 0.01 | gi\|640840007\|gb\|CP005986.1\| | 1.68975e-13 | M01232:1:000000000-AP50K:1:1101:13004:15267 | |
| *Pseudomonas simiae* | 1 | 0.01 | gi\|646231607\|gb\|CP007637.1\| | 1.28861e-89 | M01232:1:000000000-AP50K:1:1105:7226:9188 | |
| Uncultured *Curvibacter* | 1 | 0.01 | gi\|741986181\|gb\|KM603398.1\| | 4.83672e-17 | M01232:1:000000000-AP50K:1:1101:8990:7351 | |
| *Polyangium brachysporum* | 1 | 0.01 | gi\|826168461\|gb\|CP011371.1\| | 2.87179e-56 | M01232:1:000000000-AP50K:1:1108:24108:17213 | |
| *Burkholderia ambifaria* | 1 | 0.01 | gi\|171994659\|gb\|CP001026.1\| | 2.33425e-12 | M01232:1:000000000-AP50K:1:1108:20370:3233 | |
| *Aeromonas veronii* | 1 | 0.01 | gi\|328802836\|gb\|CP002607.1\| | 1.54168e-23 | M01232:1:000000000-AP50K:1:1103:20874:20124 | |
| *Bordetella bronchiseptica* | 1 | 0.01 | gi\|408445111\|emb\|HE965807.1\| | 8.7383e-17 | M01232:1:000000000-AP50K:1:1101:16030:10070 | |
| *Bacillus gaemokensis* | 1 | 0.01 | gi\|926657055\|dbj\|LC076295.1\| | 2.39633e-26 | M01232:1:000000000-AP50K:1:1105:19352:19047 | |
| *Sphingomonas sanxanigenens* | 1 | 0.01 | gi\|569540043\|gb\|CP006644.1\| | 2.48935e-27 | M01232:1:000000000-AP50K:1:1103:25091:12200 | |
| *Luteibacter jiangsuensis* | 1 | 0.01 | gi\|926663115\|ref\|NR_132709.1\| | 1.69768e-16 | M01232:1:000000000-AP50K:1:1105:18796:18324 | |
| *Comamonas sp.* | 1 | 0.01 | gi\|478895874\|gb\|KC771559.1\| | 2.87084e-115 | M01232:1:000000000-AP50K:1:1103:4605:7699 | |
| *Comamonas acidovorans* | 1 | 0.01 | gi\|3608182\|dbj\|AB009273.1\| | 3.26534e-75 | M01232:1:000000000-AP50K:1:1103:7360:21176 | |
| *Polaribacter sp.* | 1 | 0.01 | gi\|440546819\|gb\|JX272927.1\| | 1.91635e-27 | M01232:1:000000000-AP50K:1:1101:26431:14022 | |
| *Lysinibacillus sp.* | 1 | 0.01 | gi\|902947085\|gb\|KP728971.1\| | 1.53549e-71 | M01232:1:000000000-AP50K:1:1105:11731:15534 | |
| Uncultured *Bacteroidetes/chlorobi* | 1 | 0.01 | gi\|84663643\|gb\|DQ211467.2\| | 3.5341e-105 | M01232:1:000000000-AP50K:1:1108:9790:1227 | |
| *Xanthomonas translucens* | 1 | 0.01 | gi\|828451590\|gb\|CP008714.1\| | 1.12576e-26 | M01232:1:000000000-AP50K:1:1101:14729:7486 | |
| *Acidovorax avenae* | 1 | 0.01 | gi\|323371659\|gb\|CP002521.1\| | 1.27289e-47 | M01232:1:000000000-AP50K:1:1105:15660:23895 | |
| *Arthrobacter sp.* | 1 | 0.01 | gi\|767256459\|gb\|CP011005.1\| | 1.21266e-05 | M01232:1:000000000-AP50K:1:1119:24624:19176 | |
| *Pseudomonas denitrificans* | 1 | 0.01 | gi\|472247168\|gb\|CP004143.1\| | 1.23612e-09 | M01232:1:000000000-AP50K:1:1101:9871:8087 | |
| *Bosea sp.* | 1 | 0.01 | gi\|171191219\|gb\|EU373419.1\| | 1.97705e-147 | M01232:1:000000000-AP50K:1:1119:7758:6111 | |
| *Sphingomonas taxi* | 1 | 0.01 | gi\|695169020\|gb\|CP009571.1\| | 1.33667e-13 | M01232:1:000000000-AP50K:1:1104:10083:12187 | |
| *Methylobacterium populi* | 1 | 0.01 | gi\|179342784\|gb\|CP001029.1\| | 8.63565e-07 | M01232:1:000000000-AP50K:1:1101:10878:22159 | |
| Uncultured ochrobactrum | 1 | 0.01 | gi\|926657571\|dbj\|LC001074.1\| | 1.53081e-28 | M01232:1:000000000-AP50K:1:1108:19790:25004 | |
| *Intrasporangium calvum* | 1 | 0.01 | gi\|315587265\|gb\|CP002343.1\| | 3.74369e-40 | M01232:1:000000000-AP50K:1:2101:25050:4735 | |
| *Bradyrhizobium sp.* | 1 | 0.01 | gi\|146189981\|emb\|CU234118.1\| | 2.6384e-07 | M01232:1:000000000-AP50K:1:1103:12172:8824 | |
| *Xanthomonadaceae bacterium* | 1 | 0.01 | gi\|321156606\|emb\|FR774560.1\| | 7.31561e-87 | M01232:1:000000000-AP50K:1:1119:23379:4142 | |
| *Bacillus pumilus* | 1 | 0.01 | gi\|924878695\|gb\|KT624198.1\| | 2.45626e-37 | M01232:1:000000000-AP50K:1:1103:20824:18248 | |
| *Thiomonas intermedia* | 1 | 0.01 | gi\|295794626\|gb\|CP002021.1\| | 1.95876e-18 | M01232:1:000000000-AP50K:1:1101:22371:21250 | |
| *Labilithrix luteola* | 1 | 0.01 | gi\|913610804\|gb\|CP012333.1\| e | 1.83698e-06 | M01232:1:000000000-AP50K:1:1101:20154:9066 | |
| *Pseudogulbenkiania sp.* | 1 | 0.01 | gi\|345641016\|dbj\|AP012224.1\| | 3.29437e-31 | M01232:1:000000000-AP50K:1:1104:5848:18614 | |
| *Caulobacter sp.* | 1 | 0.01 | gi\|167346403\|gb\|CP000927.1\| | 1.86858e-42 | M01232:1:000000000-AP50K:1:1103:16557:4994 | |
| *Methylobacterium oryzae* | 1 | 0.01 | gi\|689271676\|gb\|CP003811.1\| | 1.76166e-19 | M01232:1:000000000-AP50K:1:1108:10675:5885 | |
| *Mycobacterium rhodesiae* | 1 | 0.01 | gi\|359817839\|gb\|CP003169.1\| | 1.43798e-39 | M01232:1:000000000-AP50K:1:1104:8915:20740 | |
| Uncultured *Sphingobium* | 1 | 0.01 | gi\|383617482\|gb\|JQ288598.1\| | 9.39564e-131 | M01232:1:000000000-AP50K:1:1119:15936:12333 | |
| *Xanthomonas axonopodis* | 1 | 0.01 | gi\|346647687\|gb\|CP002914.1\| | 2.86078e-56 | M01232:1:000000000-AP50K:1:1105:16003:14237 | |
| *Vitreoscilla stercoraria* | 1 | 0.01 | gi\|219846304\|ref\|NR_025894.1\| | 5.94979e-28 | M01232:1:000000000-AP50K:1:1105:17422:1221 | |
